# Serum Endothelin-1 Level Can Reflect the Degree of Lumbar Degeneration: A Cross-Sectional Study

**DOI:** 10.7759/cureus.59966

**Published:** 2024-05-09

**Authors:** Emine Yıldırım Uslu, Arif Gülkesen, Gurkan Akgol, Gökhan Alkan, Ahmet Kürşad Poyraz, Necip İlhan

**Affiliations:** 1 Physical Medicine and Rehabilitation, Elazığ Fethi Sekin City Hospital, Elazig, TUR; 2 Physical Medicine and Rehabilitation, Firat University, Elazig, TUR; 3 Physical Medicine and Rehabilitation, Firat University Hospital, Elazig, TUR; 4 Radiology, Firat University, Elazig, TUR; 5 Biochemistry, Firat University, Elazig, TUR

**Keywords:** intervertebral disc degeneration, biomarker, pfirrmann grading, lumbar disc herniation, endothelin-1

## Abstract

Background

Endothelin-1 (ET-1) is an agent closely associated with inflammation and has recently been recognized as a significant factor in degenerative processes. This study aimed to investigate the correlation between serum ET-1 level and radiological and clinical manifestations of lumbar disc herniation (LDH) and intervertebral disc degeneration (IDD) pathologies.

Methodology

The study was conducted with 50 healthy controls and 50 LDH patients. The pain level of the patients was analyzed with the Visual Analog Scale (VAS), and their functionality was analyzed with the Oswestry Disability Index (ODI). The disc degeneration and disc herniation grades were determined using magnetic resonance imaging. Serum ET-1 levels of the participants were measured using the enzyme-linked immunosorbent assay method.

Results

ET-1 level was significantly higher in the patient group compared to the controls (p < 0.01). A positive correlation was determined between serum ET-1 level and Pfirrmann grade in the patient group (p < 0.01). No correlation was determined between the MacNab grade, VAS, and ODI scores and ET-1 (p = 0.397, p = 0.137, and p = 0.208, respectively). There was no significant difference between the serum ET-1 levels of the patients with or without neurological deficits (p = 0.312).

Conclusions

The correlation between the serum ET-1 levels and IDD grade suggested that the former could serve as a biomarker to determine the degree of degeneration in the future. However, further research is required to determine the underlying mechanisms.

## Introduction

Lumbar pain is a global public health issue that affects about 80% of the adult population and leads to loss of quality of life and labor [1,2]. Its etiology is diverse, and the most common factor is lumbar intervertebral disc degeneration (IDD) [3]. IDD is a consequence of progressive changes introduced by aging that affect the spine [4]. IDD is characterized by changes in the cell count, phenotype, extracellular matrix (ECM) synthesis, and biomechanical properties, and can result in lumbar disc herniation (LDH), which, in turn, causes lumber pain, sciatica, and spinal instability [5,6].

Currently, IDD is often identified with magnetic resonance imaging (MRI) after it becomes symptomatic. MRI diagnosis is based on the evaluation of the changes in disc hydration, height, and contour [7]. Thus, MRI diagnosis is not possible in the early stages of degeneration, when morphological changes are not prominent. The high cost and long duration are among the limitations of MRI [8]. Novel rapid and reliable methods are needed for early IDD diagnosis. Thus, interest in biomarker research has increased recently [9-11]. Proinflammatory and inflammatory cytokines and chemokines, known to play a role in pathogenesis, have been the focus of interest in these studies [12-15].

Inflammatory mediators and signaling pathways are important in the onset and prognosis of disc degeneration [16]. It was reported that proinflammatory cytokines such as tumor necrosis factor-alpha (TNF-α), interleukin (IL)-1, IL-6, IL-17, and interferon-gamma (IFN-γ) were high in degenerating discs [17]. Inflammatory cytokines play a key role in the pathogenesis of IDD via the promotion of ECM destruction [18]. They amplify inflammatory response, neoinnervation of the disc, and release of neurotrophic factors by infiltration and activation of immune cells [12,19]. They also play a role in the synthesis of matrix metalloproteinases (MMPs), which play a primary role in collagen and proteoglycan degradation [20].

Endothelin-1 (ET-1) is a molecule closely associated with inflammation that can lead to degeneration [21]. Bellisai et al. [22] reported that ET-1 increases TNF-α synthesis in macrophages and monocytes, while Nakano et al. [23] suggested that proinflammatory cytokines increase ET-1 expression. The induction of nitric oxide synthase by ET-1 leads to excessive nitric oxide production, activation of MMP-1 and MMP-13 synthesis, reduction of the interstitial fluid flow through a vasoconstrictive effect, and acceleration of cartilage destruction by the induction of osteocyte hypoxia leading to degeneration are among the hypotheses that aimed to explain the process [24]. It can be suggested that ET-1, a significant degeneration factor, could also be associated with lumbar pathologies. This study aimed to investigate the correlation between serum ET-1 levels and radiological and clinical manifestations of LDH and IDD.

## Materials and methods

This prospective study was conducted in accordance with the Helsinki Protocol and was approved by the Ethics Committee of Firat University on September 22, 2021 (approval number: 3781). The sample size was calculated using the sample size calculating software G*Power version 3.1.9.2 (Universität Kiel, Germany). With 90% power, 0.05 level of statistical significance, and an effect size of 0.6, the sample size for each group was calculated to be 49. Written consent was obtained from all participants. Patients who presented to the Firat University Hospital’s Physical Medicine and Rehabilitation Outpatient Clinic with chronic lumbar and leg pain, dermatomal and myotome symptoms compatible with lumbar radiculopathy clinic, and MRI findings within the last year which supported LDH and were clinically compatible were included in the study. A total of fifty patients aged 18-50 years agreed to participate in the study. Patients who had no known systemic disease (cardiovascular, renal, hepatic, neurological, endocrinological, inflammatory diseases, malignancy, etc.); no history of spinal stenosis, alcohol or substance use, and axial surgery; did not use steroids or nonsteroidal anti-inflammatory drugs within the previous week, and had a body mass index (BMI) of below 40 kg/m^2^ were included in the study. A total of 50 healthy volunteers, who had no history of lumbar pain, presented to our outpatient clinic with any symptom without any pathology, and did not have any known chronic disease, were included in the control group.

The patients were examined for locomotor system functions by a physical medicine and rehabilitation physician. The femoral stretch test, straight leg raise test, Lasègue test, and neurological examination findings were recorded. The presence of muscle strength, sensory, and reflex disorders were recorded for patients with neurological deficits. The pain severity of the patients was evaluated with the Visual Analog Scale (VAS) [25], and functionality was determined with the Oswestry Disability Index (ODI) [26].

A radiologist evaluated patient MRIs. Disc degeneration level was determined using Pfirrmann grading. According to the study by Takatalo et al. [27], the total disc degeneration scores were determined for each patient at five lumbar levels. Disc herniation levels were recorded as follows: bulging, protrusion, extrusion, and sequestration based on MacNab’s disc herniation classification system [28].

Blood samples were collected from all participants between 09.00 and 11.00 am following night fasting. The systolic and diastolic blood pressures of the participants were measured twice at five-minute intervals after a 30-minute rest. When they were normotensive, 3 mL of blood was collected from the antecubital vein into tubes without an anticoagulant. The serum was separated using a 2,500 rpm centrifuge for 10 minutes. The serum was placed in Eppendorf tubes and stored at -80°C until the day of the experiment. All samples were analyzed with Sunred Human ET-1 Elisa Kit (Sunred Biological Tech. Company, catalog number: 201-12-1239, Shanghai, China), and ET-1 measurements were recorded (measurement unit: ng/L, test range: 1-300 ng/L, intra-assay CV: <10%, inter-assay CV: <12%, test sensitivity: 0.915 ng/L). ET-1 measurements were conducted using the enzyme-linked immunosorbent assay method at Firat University Faculty of Medicine Biochemistry Laboratory.

The study findings were interpreted using SPSS version 26.0 software (IBM Corp., Armonk, NY, USA). Descriptive statistics are presented as counts, percentages, and mean ± standard deviation. Intergroup comparisons were conducted using the t-test for continuous variables and the chi-square test for categorical variables. Independent factors were identified with binary logistic regression analysis. Correlations were determined using the Pearson correlation test. P-values <0.05 were accepted as statistically significant.

## Results

Gender, age, BMI, and smoking status were compared for the patient and control groups, and no statistically significant difference was determined between the groups based on these variables (p = 0.539, p = 0.100, p = 0.056, and p = 0.476, respectively). The straight leg raise test was positive in 18 (36%), the Lasègue test was positive in 14 (28%), and the femoral stretch test was positive in two (4%) patients. Neurological deficits were observed in 11 (22%) patients, including loss of muscle strength, decreased deep tendon reflexes, and hypoesthesia in the relevant dermatome in two (4%) patients; decreased deep tendon reflexes and hypoesthesia in the relevant dermatome in four (8%) patients; and hypoesthesia in the relevant dermatome in five (10%) patients. The mean VAS of the patients was 5.54 ± 1.92, and the mean ODI was 23.62 ± 9.59. As the ODI score was between 21% and 40%, the disability level of the patient group was determined as a moderate disability. ET-1 was 123.829 ± 48.909 ng/L in the patient group and 73.761 ± 47.554 ng/L in the control group. A statistically significant difference was determined between the two groups (p < 0.01) (Table 1).

**Table 1:** Demographic, clinical, and laboratory findings for the patient and control groups. P < 0.05 was accepted as significant. BMI = body mass index; VAS = Visual Analog Scale; ODI = Oswestry Disability Index; SLRS = straight leg raise test

	Patient	Control	P-value
Gender (female N, %)	32 (64%)	29 (58%)	0.539
Age (years) (mean ± SD)	40.36 ± 8.34	37.92 ± 6.20	0.100
BMI (kg/m^2^) (mean ± SD)	26.854 ± 2.503	25.762 ± 3.12	0.056
Smoking (N, %)	13 (26%)	10 (20%)	0.476
Endothelin-1 (ng/L) (mean ± SD)	123.829 ± 48.909	73.761 ± 47.554	<0.01
VAS (mean ± SD)	5.54 ± 1.92	-	-
ODI (mean ± SD)	23.62 ± 9.59	-	-
Neurological deficit (N, %)	11 (22%)	-	-
SLRS (+) (N, %)	18 (36%)	-	-
Lasègue (+) (N, %)	14 (28%)	-	-
Femoral stretch (+) (N, %)	2 (4%)	-	-

Lumbar MRI revealed diffuse bulging in 11 (22%), protruded disc in 25 (50%), extruded disc in 10 (20%), and sequestered disc in four (8%) patients based on the MacNab classification. Grade 1 disc degeneration was identified in one (2%), grade 2 was identified in eight (16%), grade 3 was identified in 17 (34%), grade 4 was identified in 12 (24%), and grade 5 was identified in 12 (24%) patients based on Pffirrmann grading. The correlation between LDH and lumbar IDD was analyzed; however, no correlation was determined in the patient group (r = 0.073, p = 0.613) (Table 2).

**Table 2:** Statistical analysis of lumbar disc herniation subtypes and disc degeneration grades. According to the Pearson test: r = 0.073, p = 0.613. P < 0.05 was accepted as significant.

	Grade 1	Grade 2	Grade 3	Grade 4	Grade 5	Total
Bulging (N, %)	-	3 (27%)	4 (36%)	4 (36%)	-	11
Protruded (N, %)	-	3 (12%)	9 (36%)	3 (12%)	10 (40%)	25
Extruded (N, %)	1 (10%)	1 (10%)	4 (40%)	3 (30%)	1 (10%)	10
Sequestered (N, %)	-	1 (25%)	-	2 (50%)	1 (25%)	4

In the regression analysis performed to evaluate the effect of patients’ age and BMI on ET-1 levels, it was determined that ET-1 was independently effective (Table 3).

**Table 3:** Regression analysis of ET-1 levels with age and BMI in patients with LDH. R^2^ (Cox-Snell) = 0.227, R^2^ (Nagelkerke) =  0.303,  p <0.001. P < 0.05 was accepted as significant. BMI = body mass index; ET-1: endothelin-1; LDH = lumbar disc herniation

	LDH		
	Exp(B)	95% CL	P-value
Constant	0.003	-	0.024
Age	1.020	0.958–1.085	0.538
BMI	1.129	0.962–1.325	0.138
ET-1	1.019	1.010–1.029	<0.001

**Figure 1:**
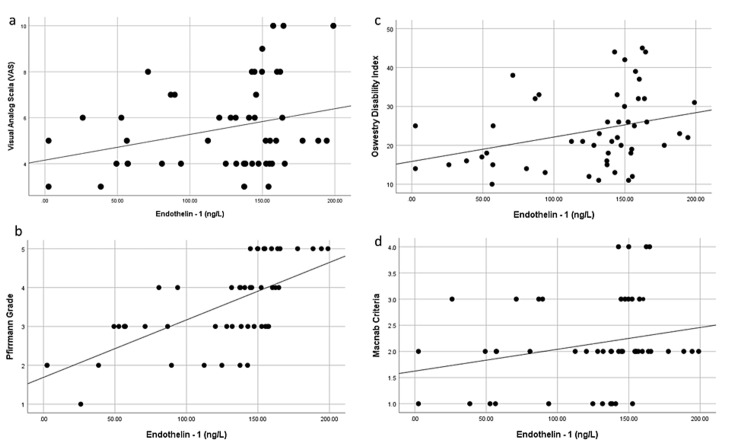
Relationship between serum ET-1 levels and VAS, ODI, Pfirrmann, and MacNab grades. (a) Correlation analysis between serum ET-1 and VAS (r = -0.213, p = 0.137). (b) Correlation analysis between serum ET-1 and ODI (r = 0.181, p = 0.208). (c) Correlation analysis between serum ET-1 and Pfirrmann grades (r = 0.662, p < 0.01). (d) Correlation analysis between serum ET-1 and MacNab grades (r = 0.237, p = 0.397). ET-1 = endothelin-1; VAS = Visual Analog Scale; ODI = Oswestry Disability Index

The correlations between ET-1 and VAS, ODI, Pfirrmann, and MacNab grades were analyzed in the patient group. There was a positive correlation between Pfirrmann grades and ET-1 (p < 0.01), while no correlations were determined between ET-1 and VAS, ODI, and MacNab grades (p = 0.137, p = 0.218, and p = 0.397, respectively) (Figure 1).

No significant difference was observed between the ET-1 levels of the patients with and without neurological deficits (Table 4).

**Table 4:** Correlation between ET-1 and neurological deficit. P < 0.05 was accepted as significant. ET-1 = endothelin-1

Neurological deficit	N	ET-1	P-value
Yes	11	137.12 ± 42.20	0.312
No	39	120.07 ± 50.50	

## Discussion

This study showed that serum ET-1 level was higher in LDH patients compared to the controls. A positive correlation was noted between serum ET-1 level and IDD grade in the patient group. These findings suggested that ET-1 could be a potential biomarker for the degree of IDD.

Mechanical overload and degeneration are the most important factors in LDH etiopathogenesis [29]. The correlation between disc degeneration and herniation was not fully elucidated, and upregulation of degenerative pathways was also identified in herniation [30]. Thus, it was suggested that degeneration could be a risk factor for herniation [4]. In a study conducted on 60 patients, Türk et al. [31] reported a positive correlation between degeneration and herniation severity. In this study, no correlation was determined between these two. This could be due to the different sample sizes and distribution.

Increasing evidence has confirmed that inflammation plays a key role in IDD pathogenesis [12,32]. ET-1 was also associated with inflammation and considered to promote proinflammatory mechanisms by increasing superoxide radical production and cytokine release. It was demonstrated to play a role in certain events, including the activation of transcription factors such as nuclear factor kappa B and stimulation of proinflammatory cytokine production such as TNF-α, IL-1, and IL-6 [33,34]. In recent years, the interest in ET-1 in degenerative pathologies has increased. Roy-Beaudry et al. [21] reported on the ET-1 expression and synthesis in human articular cartilage and synovial membrane tissues and associated it with articular cartilage degeneration. Yuan et al. [35] observed high ET-1 synthesis in degenerated endplates in a study, where they hypothesized that ET-1 could be associated with vertebral endplate degeneration based on the similarity between articular cartilage and vertebral endplates. They demonstrated that ET-1 significantly increased MMP-1 and MMP-13 release in cartilage endplates, possibly promoting ECM degradation. Zhao et al. [36] reported a correlation between serum ET-1 and the radiographic knee osteoarthritis levels. Atar et al. [37] observed that serum ET-1 levels were higher in knee and hand osteoarthritis patients when compared to the controls. Only a few studies investigated the correlations between serum ET-1 levels and LDH and IDD. In this study, serum ET-1 levels were higher in LDH patients when compared to the controls, similar to the findings reported by Goryocheva et al. [38] This finding suggested that the compression of the nerve root by a herniated disc could induce systemic ET-1 production. Furthermore, while there was no correlation between disc herniation degrees and ET-1 levels, a positive correlation was observed between the degree of disc degeneration and ET-1. Although these findings suggested that ET-1 was more associated with degeneration than herniation, whether circulating ET-1 leads to degenerative changes or is elevated due to degeneration and pain is unknown.

Neurological deficits such as decreased deep tendon reflexes, sensory deficits, and loss of muscle strength can be observed in lumbar disc hernia patients [39]. Chemical inflammation is a pathophysiological response to the compression of the neighboring neural structures by the herniated disc [40]. It could be suggested that the inflammation leads to a conduction block in nerve axons or roots; and thus, loss of sensory and/or motor functions [41]. ET-1 could play a role in perineural chemical inflammation and be associated with the development of neurological deficits. In this study, although serum ET-1 levels were higher in patients with neurological deficits when compared to those without, the difference was not statistically significant. This finding could be due to the non-homogenous distribution of the individuals in the two groups.

Pain is a prominent symptom of LDH. Inflammatory cytokines cause an immune response in the disc and lead to the release of neurotrophic factors, which, in turn, lead to discogenic pain via the sensitization of spinal nerves [42]. ET-1 could play a role in inflammation-induced pain. In a study conducted on mice, Gokin et al. [43] demonstrated that subcutaneous ET-1 molecule injection could stimulate nociceptive C fibers based on the dose and induce a pain response. ET-1 directly activates nociceptors and strengthens the effect of algogens such as capsaicin and arachidonic acid [44]. It was demonstrated that ET-1 plays a role in the pain associated with inflammation, neuropathy, cancer, and diabetic neuropathy pain [45-47]. The molecule could be associated with discogenic pain. However, in this study, no correlation was observed between the VAS scores and serum ET-1 levels of the patients. Furthermore, there was no correlation between ODI scores that indicate functional disability and serum ET-1 levels. This may be due to the difference in patients’ pain thresholds and the effect of psychogenic factors accompanying chronic low back pain on subjective pain measurements [48].

The limitations of our study include the fact that it was a cross-sectional study on a small sample, subgroup distributions were not homogeneous, ET-1 was studied only in serum and no immunohistochemical research was conducted, and pain threshold analysis was not performed.

## Conclusions

The correlation between the severity of degeneration on MRI and serum ET-1 levels in our study suggests that ET-1 may be used as a biomarker to determine the degree of degeneration in the future. Our study is the first to show the correlation between the degree of IDD and serum ET-1 level. However, further research is needed to determine the underlying mechanisms. Although serum ET-1 level could not be associated with clinical findings and functional disability levels in the present study, a correlation could be determined in future studies with larger samples.
